# A modified pharmacy provider‐led delivery model of oral HIV pre‐ and post‐exposure prophylaxis in Kenya: a pilot study extension

**DOI:** 10.1002/jia2.26467

**Published:** 2025-06-26

**Authors:** Stephanie D. Roche, Victor Omollo, Peter Mogere, Magdaline Asewe, Stephen Gakuo, Preetika Banerjee, Kendall Harkey, Monisha Sharma, Jillian Pintye, Melissa Latigo Mugambi, Parth Shah, Josephine Odoyo, Patricia Ong'wen, Daniel Were, Elizabeth A. Bukusi, Kenneth Ngure, Katrina F. Ortblad

**Affiliations:** ^1^ Public Health Sciences Division Fred Hutchinson Cancer Center Seattle Washington USA; ^2^ Centre for Microbiology Research Kenya Medical Research Institute Kisumu Kenya; ^3^ Partners in Health and Research Development Thika Kenya; ^4^ Department of Global Health University of Washington Seattle Washington USA; ^5^ Jhpiego Nairobi Kenya; ^6^ Departments of Obstetrics and Gynecology University of Washington Seattle Washington USA; ^7^ School of Public Health Jomo Kenyatta University of Agriculture and Technology Nairobi Kenya

**Keywords:** differentiated service delivery, HIV prevention, Kenya, post‐exposure prophylaxis, pre‐exposure prophylaxis, private pharmacies

## Abstract

**Introduction:**

Private pharmacies in Africa reach individuals with ongoing and periodic HIV risk, yet few countries currently leverage pharmacies as an HIV service delivery platform. We conducted a 6‐month pilot to evaluate a model for pharmacy provider‐led delivery of HIV pre‐ and post‐exposure prophylaxis (PrEP and PEP) in Kenya.

**Methods:**

At 12 private pharmacies in Kisumu and Kiambu Counties, licensed pharmacy providers initiated and managed eligible clients ≥18 years on PrEP and PEP under remote clinician supervision (NCT04558554); four of these pharmacies additionally offered sextually transmitted infection (STI) testing. PrEP/PEP clients were scheduled for follow‐up 1 month later and then quarterly (PrEP clients only). Primary outcomes included PrEP and PEP initiation and continuation during the pilot period. Client and providers rated the model across multiple constructs of acceptability and feasibility from established frameworks.

**Results:**

From January to July 2022, 1028 clients interested in PrEP, PEP and/or STI testing were screened and 829 initiated one or more service: 661 PrEP, 162 PEP and 52 STI testing. About half of clients (48%, 398/829) were male, most were unmarried (78%, 644/829) and PrEP‐naïve (89%, 737/829), and the median age was 25 years (IQR 22–31). Most PrEP clients reported inconsistent condom use (88%, 581/661) or sex with partners of unknown HIV status (70%, 460/661) in the past 6 months. Most PEP clients reported condomless sex (48%, 78/162) or a condom break (46%, 75/162) in the past 72 hours; 4% (6/162) reported sexual assault. Among PrEP clients eligible for a refill, 73% (479/658) refilled at least once and 60% (197/328) twice. Among PEP clients eligible for follow‐up, 44% (65/148) completed follow‐up HIV testing and 20% (30/148) transitioned to PrEP. Among STI clients, 19% (10/52) tested positive for gonorrhoea (*n* = 7) and/or chlamydia (*n* = 5). Most clients and providers (≥92%) found the delivery model and its implementation strategies acceptable. All providers (*n* = 12) thought it was possible to deliver PrEP and PEP at pharmacies in Kenya.

**Conclusions:**

Pharmacy PrEP/PEP delivery achieved high uptake, continuation and acceptability among eligible clients that could benefit, highlighting the potential of pharmacies to expand HIV prevention service coverage in Kenya, particularly to individuals not accessing these services at clinics.

## 1 INTRODUCTION

With shrinking donor support for HIV programmes [1], many governments in Africa are seeking strategies to sustain their HIV response, including leveraging the private sector [2, 3, 4, 5, 6]. In Kenya, one delivery channel of interest is its robust private pharmacy sector [7], which includes 7400 registered pharmacies predominantly owned and operated by the country's 11,000 licensed pharmaceutical technologists and 2600 licensed pharmacists [8, 9]. Embedded in local neighbourhoods, pharmacies have strong community reach, convenient opening hours and offer fast, discreet services [10, 11]. However, evidence is lacking on the uptake, feasibility, and acceptability of pharmacy delivery in Kenya, particularly of HIV post‐exposure prophylaxis (PEP) and STI testing.

Pharmacy delivery of PEP and STI testing alongside pre‐exposure prophylaxis (PrEP) is novel, as the current Kenya AIDS Strategic Framework only focuses on clinic‐based PEP delivery [2] and national STI guidelines continue to recommend syndromic management [12]. At Kenyan public clinics, most providers continue to treat PEP as a last‐resort emergency measure, rather than an additional prevention option for individuals with infrequent, unplanned HIV exposures [13, 14]. Early evidence from two pilots in East Africa suggest that pharmacy clientele may benefit from STI testing; among 428 adult pharmacy clients in Uganda, 11% tested positive for gonorrhoea (NG), 9% for chlamydia (CT), and 3% for syphilis [15], and among 495 female pharmacy clients age 15−24 in Kenya, 21% tested positive for CT, 6% for NG, and 3% for both [16].

From November 2020 to October 2021, our team evaluated a delivery model in which trained Kenyan pharmacy providers initiated and managed clients on PrEP under remote clinician supervision; we found high uptake among PrEP‐naïve individuals, including populations that do not frequently access health services at public clinics [17]. We now report on a 6‐month extension of this pilot in which we evaluated a modified version of the model featuring a package of implementation strategies, including PEP and STI testing. We assessed initiation and continuation, client engagement in implementation strategies, and client and provider perceptions of the model's acceptability and feasibility.

## 2 METHODS

### 2.1 Study design and setting

We conducted a single‐arm pilot evaluation (ClinicalTrials.gov NCT04558554) at 12 pharmacies evenly split between Kisumu and Kiambu Counties, with population‐level HIV prevalence of 15% and 2%, respectively [18]. We collaborated with county health officials to identify pharmacies meeting the following criteria: (1) current license and registration; (2) full‐time licensed pharmacist or pharmaceutical technologist; and (3) private consultation room—all required by Kenya's pharmacy practice guidelines [19]; (4) private bathroom (if offering STI testing services); and (5) willing to have a research assistant (RA) stationed on site Monday through Friday to conduct research activities. Since the majority of pharmacies in Kenya are independently owned, we purposely excluded company‐owned, retail chain pharmacies. To capture variation in pharmacy size, we recruited some pharmacies that served ∼100 clients per day (*n* = 4), with the remainder (*n* = 8) serving ∼50 clients per day.

### 2.2 PrEP/PEP delivery model

Our modified delivery model (Figure S1) includes six new implementation strategies, detailed in Table 1. To meet the needs of clients reporting recent high‐risk exposures to HIV or STIs, we added PEP and, at a subset of four pharmacies, STI testing. To address client discomfort discussing behaviours associated with HIV risk and undergoing HIV testing at the pharmacy, we added an option to self‐screen for HIV risk and offered free HIV self‐testing (HIVST) kits for at‐home testing. To mitigate cost barriers, we eliminated the client fee. Lastly, to increase PrEP demand, we offered PrEP clients an incentive of 100 KES ($0.80 USD) of airtime to refer peers.

**Table 1:** Package of implementation strategies deployed in the Pharm PrEP Pilot Extension to influence adoption (i.e., initiation, continuation; *client‐level)*, acceptability *(client‐ and provider‐level)*, and feasibility *(provider‐level)*

Strategy *ERIC equivalent* ^a^	Actor(s), action(s) and *dose*	Target(s)	Justification	Number of pharmacies
**Eliminate client fee** *Alter patient fees*	Research team removes ∼$3 USD fee clients paid in original pilot for pharmacy PrEP. *Dose: All study visits*.	Current and prospective PrEP clients	In original pilot, some participants said fee was a barrier to initiating and/or continuing PrEP at the pharmacy.	12 pharmacies
**Offer self‐screening for HIV risk behaviours** *Intervene with patients to enhance uptake and adherence*	Pharmacy provider gives clients option to self‐administer HIV risk screening tool. Client answers HIV risk questions. Pharmacy provider reviews responses. *Dose: Once per study visit*	Current and prospective PrEP clients	Several implementation studies in Kenya, including the original Pharm PrEP pilot, have documented client discomfort with provider‐administered HIV risk screening; some clients have expressed interest in administering the screening tool themselves.	12 pharmacies
**Offer optional HIVST** *Intervene with patients to enhance uptake and adherence*	Pharmacy providers give clients option to take home a blood‐ or oral‐fluid HIVST. *Dose: Once per study visit*	Current and prospective PrEP clients	In the original pilot, some clients declined or were hesitant to enrol because they were not comfortable undergoing HIV testing at the pharmacy (required for PrEP initiation). Offering such clients a free HIVST kit to learn their HIV status on their own first might increase their willingness to undergo HIV testing with a pharmacy provider.	12 pharmacies
**Offer free PEP services** *Increase demand*	Research team trains pharmacy providers to deliver PEP and procures commodities. Pharmacy providers offer PEP to eligible clients. *Dose: One‐time offer*	Pharmacy clients reporting recent HIV exposure	In the original pilot, nearly half of female clients reported recurrent emergency contraception use, and clients reporting a recent exposure to HIV had to be referred to clinics for PEP. Making PEP available for free at pharmacies may fulfil a need for this service.	12 pharmacies
**Offer free STI testing services** ^b^ *Increase demand*	Research team trains pharmacy providers to deliver STI testing and procures commodities. Pharmacy providers offer free STI testing, with optional PrEP screening, to clients seeking STI testing or treatment. *Dose: One‐time offer*	Clients seeking STI testing or treatment services	Kenyans commonly seek STI treatment at pharmacies; several studies have found high STI prevalence among Kenyan PrEP users, especially AGYW. Adding STI testing to these settings may help identify potential PrEP candidates and engage them in PrEP services.	Subset of four pharmacies
**Incentivize peer referral** ^c^ *Increase demand*	Research assistants introduce peer referral concept to PrEP clients at month 1 follow‐up visit. PrEP clients refer peers (5 max) to pharmacy for PrEP/PEP screening and receive ∼$0.80 USD airtime for each referred peer who completes screening. *Dose: One‐time offer*	Peers of enrolled study participants	In the original pilot, clients commonly reported learning about pharmacy PrEP via informal word‐of‐mouth referral. PrEP clients may know others in their social networks who engage in similar behaviours and could benefit from PrEP. Incentivizing PrEP clients to tell peers about PrEP and encourage them to undergo screening at the pharmacy may enhance adoption, especially among AGYW.	12 pharmacies

Abbreviations: AGYW, adolescent girls and young women; HIVST, HIV self‐testing; PEP, post‐exposure prophylaxis; PrEP, pre‐exposure prophylaxis; USD, US Dollars.

^a^ From Powell et al.’s Expert Recommendations for Implementing Change (ERIC) compilation of discrete implementation strategies.

^b^ Urine‐based testing for *C. trachomatis* and *N. gonorrhoeae* implemented at a subset of four pharmacies. Samples are courier‐delivered to an off‐site laboratory. Clients with positive test results are notified via phone call and offered free treatment.

^c^ Participants receive incentive regardless of their peer's screening outcome or decision to enrol in study.

Pharmacy providers attended a 2‐day, in‐person training covering PrEP/PEP eligibility screening; counselling on HIV risk and adherence; assisting with HIVST; consulting a remote clinician for support and/or referrals; dispensing PrEP/PEP; and supporting self‐sampling for STI testing. Throughout, RAs stationed at the pharmacy provided technical assistance, as needed.

### 2.3 Participants

We enrolled pharmacy clients and providers. Eligible clients were ≥18 years and met the criteria on a standardized checklist (detailed below). Eligible providers were ≥18 years and willing to deliver study services. We trained pharmacy providers to recruit clients seeking sexual and reproductive health products and to display posters promoting PrEP and PEP.

The Kenya Scientific Ethics Review Unit and Institutional Review Board of the University of Washington approved this study. Participants provided written informed consent and received 500 KES (∼$4.50 USD) per survey completed. Pharmacy owners received ∼12,500 KES (∼$109 USD) monthly for their time spent delivering PrEP/PEP and use of their space and utilities by RAs; this amount was decided through consultation with pharmacy owners.

### 2.4 Procedures

Using a prescribing checklist (Figure S2), pharmacy providers conducted an HIV risk assessment, medical safety assessment, HIV testing, and drug dispensing. A remote clinician oversaw checklist implementation and was available for consultation 24/7 via phone call or SMS.

#### 2.4.1 PrEP/PEP eligibility assessment

For clients seeking PEP, providers confirmed that their potential exposure to HIV was high‐risk (e.g., condom break, sexual assault, shared needles) and occurred within the past 72 hours. For clients seeking PrEP, providers assessed ongoing HIV risk using a 12‐item modified version of Kenya's Risk Assessment Screening Tool (RAST) [20], which asks about engagement in select behaviours (e.g., transactional sex) in the past 6 months. Clients had the option to self‐administer a paper version of the RAST for subsequent provider review.

Next, providers screened potential PrEP clients for signs of acute HIV acquisition and a history of kidney disease, liver disease, and diabetes—conditions that could contraindicate drug safety—and referred clients reporting these to nearby public clinics. Serum creatinine level and hepatitis B testing were not conducted, as national guidelines advise against delaying PrEP initiation if these tests are unavailable [21].

Clients then completed provider‐assisted blood‐based HIVST (Mylan Pharmaceuticals Private Limited, Hyderabad, India). Clients who tested HIV‐negative could receive same‐day drug dispensing; clients who tested HIV‐positive were referred to nearby public clinics for confirmatory testing.

#### 2.4.2 Dispensing and follow‐up

PrEP clients received a 1‐month supply of daily oral PrEP at initiation and 3‐month supply (i.e., 90‐day refill) at each follow‐up; PEP clients received a 28‐day supply of daily oral PEP. Providers scheduled PrEP/PEP clients for follow‐up visits 1 month later and quarterly thereafter (PrEP clients only) to screen for severe side effects and to complete provider‐assisted HIVST; clients who initiated PrEP/PEP in the final month of the study were referred to nearby clinics for follow‐up. At their first follow‐up visit, PrEP clients could opt to receive up to five referral slips to distribute to peers; for each referred peer who underwent PrEP/PEP screening at the pharmacy, the referring client received the incentive. All PrEP/PEP drugs were provided to pharmacies free of charge from government stock, in line with Kenya's Private Sector Engagement Framework [3].

#### 2.4.3 STI testing

At the four pharmacies providing STI testing, providers offered this service to clients ≥18 years who came to the pharmacy seeking STI testing or treatment. This service was not otherwise advertised in the pharmacy. Self‐collected urine samples were courier‐delivered same day to a nearby research lab for *C. trachomatis* and *N. gonorrhoeae* testing (Cepheid GeneXpert, Sunnyvale, USA). Within 1 day of sample collection, the study's remote clinician called clients who tested positive and issued an antibiotic prescription that could be filled at a study pharmacy for free.

### 2.5 Data collection

The prescribing checklist was completed on paper by pharmacy providers at each visit and entered by RAs into CommCare (Dimagi, Cambridge, USA)—an electronic data collection platform. Also, in CommCare, RAs administered client surveys at the end of each pharmacy visit and provider surveys at study baseline and monthly.

### 2.6 Study outcomes

#### 2.6.1 Utilization outcomes

Our primary outcomes were PrEP initiation and continuation; secondary outcomes included PEP initiation, HIV testing at PEP follow‐up, PEP‐to‐PrEP transition, and STI prevalence. Clients initiated PrEP or PEP if they completed dispensing and continued PrEP if they completed refilling at least once. Clients transitioned from PEP to PrEP if they completed PrEP dispensing following PEP dispensing. We assessed PrEP and PEP continuation among clients who initiated these services more than 1 month prior to study endline and thus were eligible for follow‐up during the study period. PrEP clients who refilled PrEP, and PEP clients who completed follow‐up HIV testing at a study pharmacy within 15 days of their scheduled visit, were categorized as returning “on‐time.” Additionally, we assessed the timing of pharmacy PrEP/PEP visits and client engagement in STI testing, self‐screening for HIV risk, free initial HIVST, and incentivized peer referral.

#### 2.6.2 Implementation outcomes

We captured client and provider perceptions of the model and its implementation strategies. We assessed different constructs of acceptability (e.g., affective attitude, burden) based on the Theoretical Framework of Acceptability [22], presenting participants with statements tailored to each implementation strategy. We assessed feasibility using two items from the Feasibility of Implementation Measure [23]. All items used a 5‐point response scale ranging from “completely disagree” (1) to “completely agree” (5).

### 2.7 Analysis

We report outcomes descriptively using summary statistics. To understand if participants returned on time, we plotted the percentage of participants who returned for follow‐up over time and calculated median days from initiation to follow‐up. We report continuation outcomes for the following subgroups: men <25, men ≥25, women <25 and women ≥25 years. To assess for statistically significant differences (*p*<0.05) in outcomes between subgroups, we conducted Chi‐squared tests. For implementation outcomes, we decided a priori that services/strategies would be considered acceptable or feasible if ≥80% of participants “agreed” or “completely agreed” with a statement [24]. We conducted analyses in R (version 2023.03.1).

## 3 RESULTS

**Figure 1:**
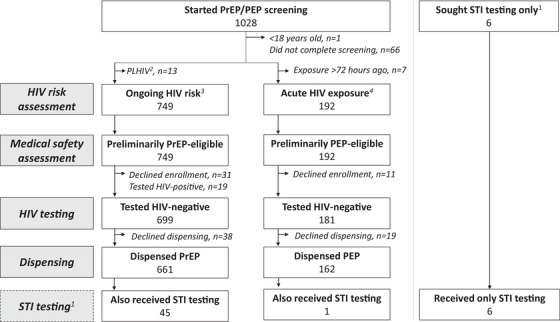
**Participant flow chart**. Abbreviations: PEP, post‐exposure prophylaxis; PrEP, pre‐exposure prophylaxis; STI, sexually transmitted infections. ^1^STI testing offered at four study pharmacies; clients seeking STI testing were given the option to screen for PrEP/PEP eligibility; ^2^Self‐identified as a person living with HIV (PLHIV); ^3^Self‐reported behaviors in past 6 months; ^4^Self‐reported a high‐risk exposure in past 72 hours.

From January to July 2022, 1028 clients began PrEP/PEP eligibility screening (Figure 1). Among 880 clients who tested HIV‐negative, 699 (79%) were determined eligible for PrEP and 181 (21%) for PEP. Nineteen clients tested HIV‐positive and were referred to public clinics. In the four pharmacies offering STI testing, 52 clients received this service. All providers (*n* = 12) completed surveys.

### 3.1 PrEP and PEP initiation

PrEP initiation among eligible clients was 95% (661/699) and PEP initiation was 90% (162/181). Whereas the most common day for PEP initiation was Monday (37%, 60/162), PrEP initiations were evenly spread from Monday to Friday, Figure S3. The average monthly number of PrEP clients initiated at each pharmacy was 9.2 (standard deviation [SD] 4.5) and PEP clients was 2.3 (SD 2.1). Among PrEP/PEP clients, roughly half were men (48%, 394/823), <25 years (48%, 393/823) and had completed secondary school (50%, 408/823) (Table 2). Additionally, most were unmarried (78%, 640/823), PrEP‐naïve (89%, 731/823) and said pharmacies are their first stop for non‐urgent healthcare needs (75%, 619/823). Men comprised a significantly higher proportion of PEP (58%, 94/162) versus PrEP (45%, 300/661) clients (*p*<0.01).

**Table 2:** Demographic characteristics of pharmacy clients and providers who received or delivered PrEP, PEP and/or STI testing at enrolment

Characteristic	PrEP clients *N* = 661	PEP clients *N* = 162	STI testing clients^a^ *N* = 52	Providers *N* = 12
**Male**	300 (45%)	94 (58%)	18 (35%)	5 (42%)
**Age**, median (IQR)	25 (22−31)	25 (22−29)	31 (26−34)	37 (33−39)
*<25 years*	314 (48%)	79 (49%)	9 (17%)	0 (0%)
**Ever attended school**	647 (98%)	159 (98%)	49 (94%)	−
**Highest level of school attended**				
*Primary*	145 (22%)	11 (7%)	18 (35%)	0 (0%)
*Secondary*	329 (50%)	79 (49%)	11 (21%)	0 (0%)
*Post‐secondary*	136 (21%)	69 (43%)	9 (17%)	12 (100%)
**Unmarried**	506 (77%)	134 (83%)	11 (21%)	
**Monthly household income** in Kenyan shillings, median (IQR)^b^	10,000 (5000−20,000)	15,000 (722−30,000)	10,000 (5000−15,500)	
**Pharmacy is first stop** for non‐urgent healthcare	541 (82%)	78 (48%)	43 (83%)	
Pharmacy visits per month, median (IQR)	1 (1−1)	2 (1−2)	1 (1−1)	
**Emergency contraception use,** past 6 months​	74 (11%)	37 (23%)	8 (15%)	
**Ever tested for HIV**	605 (92%)	132 (81%)	48 (92%)	
*Months since last HIV test, median (IQR)*	6 (3−12)	5 (3−12)	5 (2−12)	
**HIV risk behaviours, past 6 months** ^c^				
*Inconsistent condom use*	581 (88%)		45 (87%)	
*Partner(s) HIV status unknown*	460 (70%)		40 (77%)	
*Multiple sex partners*	420 (64%)		39 (75%)	
*Sex with drugs/alcohol*	265 (40%)		26 (50%)	
*Transactional sex*	232 (35%)		35 (67%)	
*Partner living with HIV*	65 (10%)		4 (8%)	
*Recent STI*	128 (19%)		32 (62%)	
**Recent HIV exposure, past 72 hours** ^d^				
*Unprotected sex and partner status unknown*		78 (48%)		
*Condom break*		75 (46%)		
*Sexual assault*		6 (4%)		
*Other*		3 (2%)		
**PrEP awareness​**				
*Had heard of PrEP prior to enrolling*​	573 (87%)	103 (64%)	44 (85%)	
*Knows someone who takes PrEP*​	303 (46%)	18 (11%)	33 (63%)	
*Participated in original pilot*	10 (2%)	0 (0%)		
**Prior PrEP use**	89 (13%)	3 (2%)	12 (23%)	
**How heard about pharmacy PrEP/PEP** ^e^				
*From pharmacy provider*	460 (70%)	63 (39%)		
*Other word‐of‐mouth*	366 (55%)	31 (19%)		
*Saw poster at pharmacy*	135 (20%)	27 (17%)		
*Referral from nearby pharmacy*	14 (2%)	45 (28%)		
*Referral from nearby clinic*	13 (2%)	17 (10%)		
**Came to pharmacy seeking PrEP/PEP**	391 (59%)	136 (84%)		
**County where enrolled**				
*Kisumu*	454 (69%)	43 (27%)	39 (75%)	6 (50%)
*Kiambu*	207 (31%)	119 (73%)	13 (25%)	6 (50%)

Abbreviations: IQR, interquartile range; PEP, post‐exposure prophylaxis; PrEP, pre‐exposure prophylaxis; STI, sexually transmitted infections.

^a^ Includes 45 clients who also received PrEP and one client who also received PEP.

^b^ USD equivalent is $86.5 (43.2−173). Converted from KES to USD using conversion rate averaged from 1/2022 to 7/2022 ($1 USD = $115.6 KES); https://www.exchangerates.org.uk/USD‐KES‐spot‐exchange‐rates‐history‐2022.html.

^c^ Asked only of clients who did not report one or more potential exposures to HIV in past 72 hours; percentages for clients who underwent STI testing are out of a denominator of 45.

^d^ To qualify for PEP, client had to report experiencing within the past 72 hours a potential exposure to HIV that was “of high risk type” and involved “high risk material,” as defined by Kenya national PEP guidelines.

^e^ Select all that apply question.

Among PrEP clients, the most common behaviours associated with HIV acquisition risk in the past 6 months were inconsistent condom use (88%, 581/661), sex partner(s) of unknown HIV status (70%, 460/661), and multiple sex partners (64%, 420/661); only 10% (65/661) reported having a sex partner living with HIV. Among PEP clients, the most common potential HIV exposures were condomless sex with a partner of unknown HIV status (48%, 78/162), and condom break (46%, 75/162); 4% of PEP clients (6/162) reported potential exposure from sexual assault.

### 3.2 PrEP and PEP continuation

**Figure 2:**
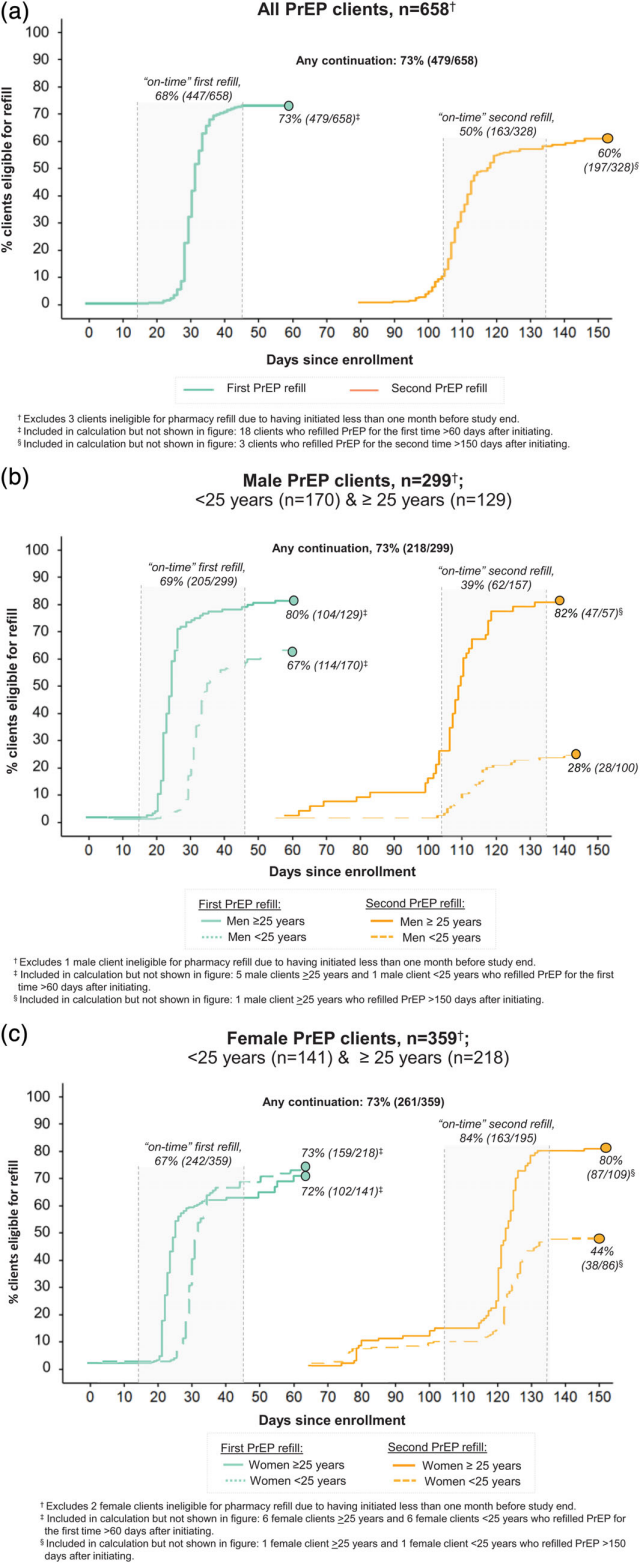
**Continuation of pharmacy PrEP services over the pilot duration**. PrEP continuation among (a) all PrEP clients; (b) male PrEP clients <25 years old and >25 years old; and (b) female PrEP clients <25 years old and >25 years old. Abbreviation: PrEP, pre‐exposure prophylaxis.

Over an observation period of 2464 person‐months, no PrEP clients seroconverted or switched to PEP. Among PrEP clients eligible for follow‐up, 73% (479/658) completed their first refill and 60% (197/328) their second refill (Figure 2a). Most PrEP continuation occurred on time: 93% (447/479) of first refills and 83% (163/197) of second refills. Median time from initiation to first refill was 30 days (interquartile range [IQR] 28–33) and to second refill was 120 days (IQR 115–125). Refilling PrEP twice was significantly higher among men ≥25 years (82%, 47/57) versus men under 25 (28%, 28/100; *p*<0.01) (Figure 2b).

**Figure 3:**
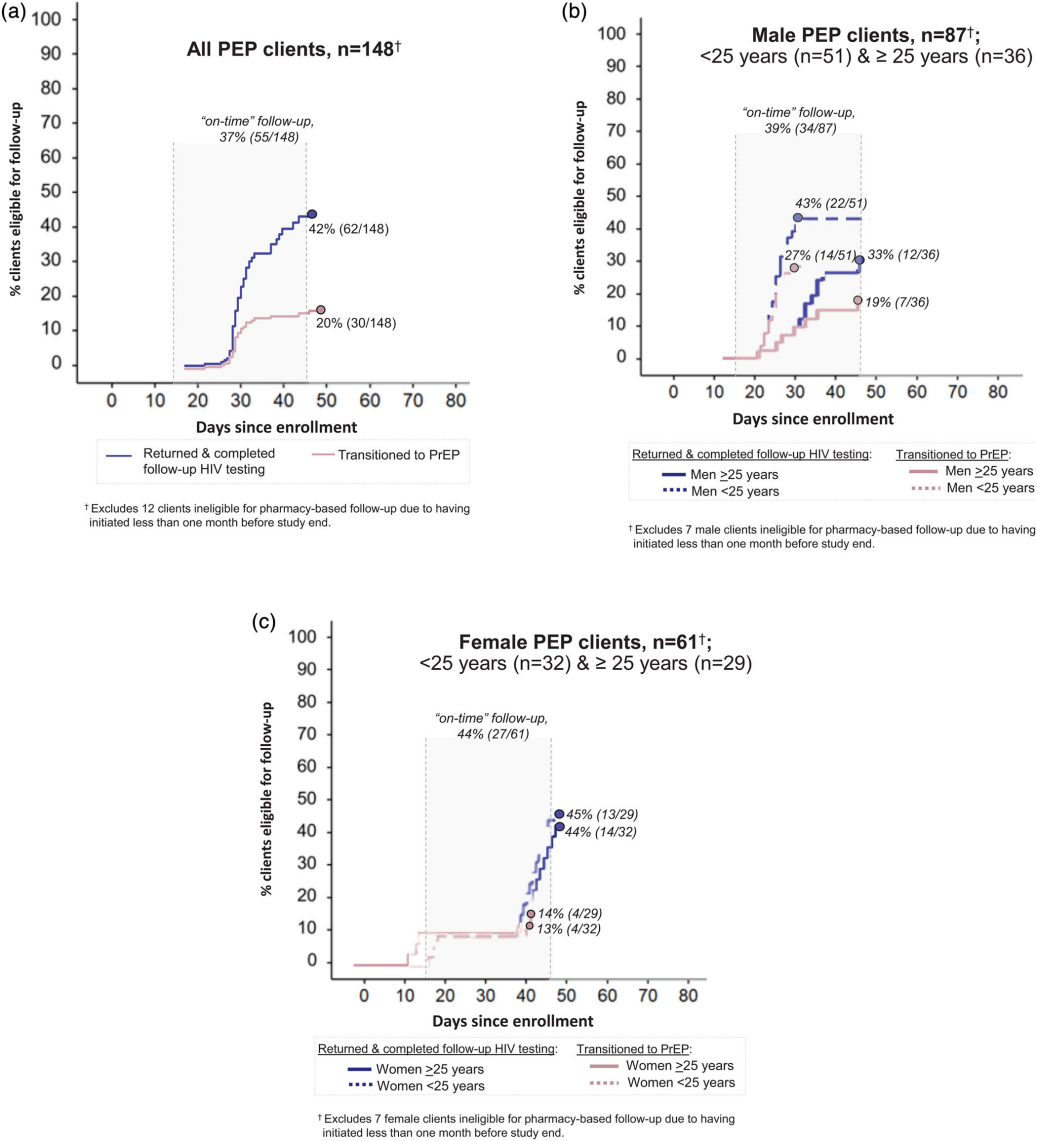
**Completion of HIV testing at PEP follow‐up visit and PEP to PrEP transition over the pilot duration**. Completion of HIV testing at pharmacy‐based follow‐up visit and PEP‐to‐PrEP transition among (a) all PEP clients; (b) male PEP clients <25 years old and >25 years old; and (b) female PEP clients <25 years old and >25 years old. Abbreviations: PEP, post‐exposure prophylaxis; PrEP, pre‐exposure prophylaxis.

Over 536 person‐months of observation, no PEP clients seroconverted or had repeat PEP use. Among PEP clients eligible for follow‐up, 42% (62/148) completed follow‐up HIV testing and 20% (30/148) transitioned to PrEP (Figure 3a). Among those who returned for follow‐up, most (85%, 55/65) did so on time. Median time from initiation to follow‐up was 30 days (IQR: 28–34). Among PEP clients who declined PrEP eligibility screening at follow‐up and completed surveys, common reasons for not transitioning to PrEP included no persistent HIV risk (77%, 10/13), anticipated disapproval from sex partners or family members (38%, 5/13), disinterest in a daily pill (31%, 4/13), and needing more time to decide (15%, 2/13). We did not find any significant differences by age and sex in the proportion of PEP clients who completed follow‐up HIV testing or transitioned from PEP to PrEP (Figure 3b,c).

### 3.3 STI testing

At the four pharmacies offering STI testing, 18% (52/289) of enrolled participants received this service (Figure 1). Uptake of STI testing varied by pharmacy, with over half (54%, 28/52) of STI testing clients enrolling at a single pharmacy (located near bars and sex‐on‐premises venues) and only 4% (2/52) at another pharmacy. Most clients who underwent STI testing (88%, 46/52) opted to screen for PrEP/PEP eligibility on the same day: 45 initiated PrEP and one PEP. STI prevalence among those who tested was 19% (10/52): five tested positive for gonorrhoea only, three for chlamydia only, and two for both. (For results by client sex, see Table S1.) All 10 clients who tested positive for an STI also completed STI treatment.

### 3.4 Implementation strategies and outcome assessment

Client engagement in opt‐in implementation strategies varied. Of the 476 PrEP clients who returned for follow‐up, 15% (72/476) successfully referred one or more peers to a study pharmacy; 16% (132/829) of participants were referred to a pharmacy by an enrolled peer, and most referred clients (80%, 106/132) characterized the person who referred them as “a close friend.” Among PrEP clients, nearly one‐fifth (18%, 120/661) opted to self‐screen for HIV risk. Few (<1%, 9/961) prospective PrEP/PEP clients who completed the HIV risk and medical safety assessments opted to receive a free HIVST for initial at‐home testing.

Client acceptability of the implementation strategies was high among those who engaged. Almost all strategy‐specific client acceptability assessments (20/21) reached our prespecified threshold of ≥80% agreement (Table 3); the one exception was clients’ assessment of intervention burden for PEP, which only achieved 73% agreement.

**Table 3:** Client and provider perceptions of acceptability and feasibility of implementation strategies they experienced or delivered

	Implementation strategy^a^													
							Opt‐in strategies							
	PEP services		STI testing (4 pharmacies)		Free PrEP		Incentivized peer referral		Initial HIV self‐test		Self‐screening for HIV risk		Overall acceptability of pharmacy‐based PrEP delivery	
	Clients *N* = 158^b^	Providers *N* = 12	Clients *N* = 51^b^	Providers *N* = 4	Clients *N* = 654^b^	Providers *N* = 12	Clients *N* = 132^c^	Providers *N* = 12	Clients *N* = 9	Providers *N* = 12	Clients *N* = 120	Providers *N* = 12	Clients *N* = 654^b^	Providers *N* = 12
**Acceptability constructs**: from the Theoretical Framework of Acceptability^d^														
Intervention coherence: Thinks the strategy is a good way to engage individuals at risk of HIV^e^	156 (99%)	12 (100%)	50 (98%)	4 (100%)	654 (100%)	12 (100%)	132 (100%)	11 (92%)	9 (100%)	11 (92%)	118 (98%)	12 (100%)	651 (99%)	12 (100%)
Affective attitude: Liked engaging in/delivering the strategy	158 (100%)	12 (100%)	50 (98%)	4 (100%)	651 (99%)	12 (100%)	132 (100%)	10 (83%)	9 (100%)	9 (75%)	120 (100%)	12 (100%)	647 (99%)	12 (100%)
Self‐efficacy: Confident in their ability to engage in the strategy	157 (99%)	11 (92%)	50 (98%)	4 (100%)	644 (98%)	‐	128 (97%)	‐	‐	‐	119 (99%)	12 (100%)	646 (99%)	12 (100%)
Burden: Strategy was ***hard*** to engage in/deliver	42 (27%)	0 (0%)	5 (10%)	0 (0%)	‐	‐	2 (2%)	‐	‐	‐	10 (8%)	1 (8%)	31 (5%)	0 (0%)
Ethicality: Strategy ***interferes*** with their other priorities	‐	0 (0%)	‐	0 (0%)	‐	0 (0%)	‐	‐	‐	‐	‐	0 (0%)	‐	0 (0%)
**Feasibility constructs**: from the Feasibility of Implementation Measure^f^														
Seems possible to implement *(with MOH help)* ^g^	‐	12 (100%)	‐	4 (100%)	‐	11 (92%)^‡‡^	‐	11 (92%)^‡‡^	‐	12 (100%)^‡‡^	‐	11 (92%)	‐	12 (100%)
Seems ***hard*** to implement in Kenya *(without MOH help)* ^h^ *(even with MOH help)* ^i^	‐	0 (0%)	‐	0 (0%)	‐	0 (0%)^¶¶^	‐	4 (33%)*^§§^*	‐	5 (41%)*^§§^*	‐	1 (8%)	‐	1 (8%)

Abbreviations: Ministry of Health (MOH); pre‐exposure prophylaxis (PrEP); post‐exposure prophylaxis (PEP)

^a^ Acceptability was only assessed among clients who received the indicated service or engaged in the indicated opt‐in implementation strategy. Clients indicated level of agreement on a 5‐point Likert scale ranging from “completely disagree” to “completely agree”. We present the number who “agreed” or “completely agreed” with each statement.

^b^ N is less than the total number of clients that received the indicated service due to 12 missing surveys: 4 from PEP clients, 1 from an STI testing client, and 7 from PrEP clients.

^c^ We assessed acceptability of incentivized peer referral only among clients who were referred to the pharmacy by an enrolled participant. We did not assess it among clients who engaged in this strategy only as a referrer.

^d^ Statements were derived from Sekhon et al.’s Theoretical Framework of Acceptability.

^e^ We tailored statements for assessing perceptions of intervention coherence to the objective of each implementation strategy as follows: Pharmacy‐based PEP services: “[…] is a good way to reach people who may have been recently exposed to HIV”; Pharmacy‐based STI testing: “[…] is a good way to help people stay healthy”; Free PrEP: “[…] is a good way to get people who are at risk of HIV to take PrEP”; Incentivized peer referral: “[…] is a good way to connect people to PrEP”; Initial at‐home HIV self‐test: “[…] is a good way to help clients feel comfortable to get tested later at the pharmacy”; Self‐screening for HIV risk: “[…] is a good way to help clients feel comfortable and complete the PrEP screening process”.

^f^ Feasibility statements were derived from Weiner et al.’s Feasibility of Implementation Measure. Clients answered using the same 5‐point Likert scale.

^g^ For these strategies, the statement posited a scenario in which the strategy was implemented with the help of the Ministry of Health as follows: Free PrEP: “If the Ministry of Health provides pharmacies with the PrEP drug, HIV self‐test kits, and some compensation for delivering PrEP, it seems possible for pharmacies in Kenya to deliver free PrEP to clients”; Incentivized Peer Referral: “It seems possible for pharmacies to implement Peer Referral in Kenya with the help of the Ministry of Health”; Initial at‐home HIVST: “It seems possible for pharmacies in Kenya to give prospective PrEP clients a free HIV self‐test kit with the help of the Ministry of Health.”

^h^ For these strategies, the statement posited a scenario in which the strategy was implemented without the help of the Ministry of Health as follows: Incentivized Peer Referral: “Peer Referral seems hard to do in Kenya without the help of the Ministry of Health”; Initial at‐home HIVST: “It seems like it would be hard for pharmacies in Kenya to give prospective PrEP clients a free HIV self‐test kit without the help of the Ministry of Health.”

^i^ For this strategy, the statement specified MOH help: “Even if the Ministry of Health provided pharmacies with the PrEP drug, HIV self‐test kits, and some compensation for delivering PrEP, it still seems like it would be hard for pharmacies in Kenya to deliver free PrEP to clients.”

Providers also rated each strategy's acceptability and feasibility as high, with 39 of 41 acceptability/feasibility assessments reaching the ≥80% agreement threshold. Three exceptions were providers’ affective attitude towards offering clients free HIVSTs (75% agreement) and concerns about ease of implementing free HIVSTs (59% agreement) and incentivized peer referral (67% agreement) without ministry of health (MOH) assistance.

For the overall PrEP delivery model, ≥95% of clients and providers found the model acceptable, and ≥92% of providers thought pharmacy PrEP delivery was feasible.

## 4 DISCUSSION

In this 6‐month pilot study, we evaluated a model of pharmacy provider‐led PrEP and PEP delivery in Kenya and observed high PrEP/PEP initiation and continuation and positive client and provider perceptions. Most participants were PrEP‐naïve yet had asubstantial ongoing risk of HIV acquisition, highlighting the potential of private pharmacies to reach individuals with HIV risk who are not reached by traditional clinic‐based programmes. PrEP continuation was high, suggesting that the implementation strategies added to the model supported client engagement.

Using pharmacies’ existing staff and infrastructure, this model achieved PrEP outcomes that match or exceed those of several recent PrEP implementation projects in Kenya. The monthly PrEP initiation rate at our 12 study pharmacies (∼9.2 initiations) surpassed those observed at 25 clinics in the Partners Scale‐up Project (∼7.5 initiations) [25] and 93 clinics in the Jilinde programme (∼6.5 initiations) [26]. Additionally, the clients reached at pharmacies in this study—particularly those <25 years, unmarried, and not in known HIV serodifferent relationships—are often underrepresented in clinic‐based PrEP programmes, where such subgroups typically comprise <20% of all PrEP clients [25]. This suggests that private pharmacies in Kenya may have different catchment populations than public clinics and that expanding PrEP services to private pharmacies might increase PrEP coverage, especially in counties with high HIV burden and public awareness of PrEP, like Kisumu County, where ∼70% of this pilot's PrEP clients enrolled. Finally, PrEP continuation in this study (72%) exceeded that of our original pilot (53%) [17] and of clinic‐based PrEP programmes in Kenya (where continuation rarely exceeds 50%) [25, 27, 28]. Possible contributing factors include the elimination of client fees and the availability of PEP, which allowed clients to initiate a biomedical prevention service better suited for their needs and appropriately discontinue if their HIV risk was not persistent.

To our knowledge, this is the first study to evaluate pharmacy provider‐led PEP delivery in Africa. Our findings demonstrate a considerable need for PEP and the value of co‐locating PrEP and PEP delivery to serve clients with dynamic HIV acquisition risk [29]. In Kenya, obtaining PEP at public clinics can be challenging due to limited opening hours, stock‐outs and low provider PEP knowledge [30, 31, 32, 33]. Recently recommended by the World Health Organization [34], community‐based PEP delivery has the potential to help clients circumvent these barriers and meet the needs of those unwilling to access PEP at clinics [13, 35]. In our study, most clients sought PEP following engagement in condomless sex over the weekend, highlighting the important role pharmacies could play in delivering this time‐sensitive service [35]. Our findings also indicate that pharmacies may reach a different subset of the PEP‐eligible population than public clinics; compared to 124 clients who initiated PEP at public clinics in a recent SEARCH pilot [30], a greater proportion of PEP clients in our study were <25 years (49% vs. 24%) and unmarried (83% vs. 42%).

Our study highlights the importance of PEP as an HIV prevention choice. For some clients, PEP may serve as an “on‐ramp” to PrEP, as illustrated by the 20% of PEP clients who opted to transition to PrEP. Other clients, however, may not want or need PrEP, especially if their potential exposures to HIV are infrequent or if they find adhering to a daily pill regimen difficult [31, 33]. Client preference for PEP over PrEP might also be common in areas with relatively low HIV burden—one possible explanation for why we observed substantially higher PEP uptake at pharmacies in Kiambu versus Kisumu County. A key area for improvement for this delivery model, however, is PEP follow‐up, as over half of PEP clients did not return. Additional research is needed to identify implementation strategies that might support follow‐up HIV testing in this population, such as dispensing PEP with an HIVST kit [36] or deploying community health workers for home‐based testing [30].

Most participants engaged in one or more implementation strategies and found them acceptable, suggesting their potential to enhance implementation in real‐world pharmacy settings. The uptake of STI testing was modest, possibly due to its lack of advertisement and/or provider hesitation to offer this service, especially to clients willing to pay for STI treatment without testing. Among clients who tested, STI prevalence was high (19%) and most (88%) clients also initiated PrEP/PEP, demonstrating the potential for STI testing to serve as a bridge to HIV prevention services. The type of STI testing conducted in this study—automated, real‐time PCR‐based nucleic acid amplification tests—may be difficult to implement at scale due to cost and logistical barriers (e.g., need for off‐site processing); however, new and forthcoming point‐of‐care rapid tests could potentially mitigate these challenges in the future [15].

Pharmacy PrEP and PEP initiations in this pilot were likely facilitated by the free cost to clients (previously 300 KES [∼$3 USD] per visit in the original pilot); incentivized peer referral, which has had similar success for engaging clients in other health services [37, 38]; and self‐screening for HIV risk, a strategy found to increase HIV testing rates in other populations [39]. Provider engagement in delivery was also likely influenced by the monthly compensation they received. Additional research is needed to assess the effect of different cost‐sharing options (e.g., sliding scale fees; private or national health insurance coverage) that might increase the scalability of this package of implementation strategies, or a subset thereof. Few clients engaged in HIVST. Possible reasons for this include inconvenience (e.g., time/cost associated with a second trip to the pharmacy for PrEP/PEP); selection bias, with clients hesitant to HIV test at a pharmacy also hesitant to test at home; and fidelity issues, such as providers forgetting to offer the free HIVSTs or opting to instead sell HIVSTs from their pharmacy's stock.

Our study has limitations. Since we only tested this model at 12 purposively selected pharmacies in two counties, our findings are not generalizable to other pharmacy settings. Our study design—a single‐arm pilot that only enrolled individuals interested in PrEP/PEP—precludes our ability to discern the effect of each implementation strategy on PrEP/PEP uptake or continuation. If pharmacy delivery becomes the standard of care, future studies could build upon this work and leverage factorial designs [40] to determine which strategies are most effective. Since our study did not include a control group, we cannot determine if the intervention led to greater initiations and/or continuation than the standard of care: pharmacy‐based screening and referral to clinics; an ongoing cluster randomized controlled trial in Kenya is investigating this question [41]. We did not assess fidelity; thus, suboptimal delivery may have influenced some outcomes. For PEP clients, we did not collect information on number of hours since potential HIV exposure. Our study's short duration may have benefited our PrEP initiation outcome by capturing initial excitement among providers and clients; with a longer observation period, we might have observed a plateau in new initiations (e.g., due to saturation and/or provider burn‐out). Since we did not assess outcomes among clients who never initiated PrEP/PEP or never returned for follow‐up, we do not know whether or how the delivery model influenced these decisions and cannot assess factors associated with initiation. Lastly, our understanding of client and provider experiences with the model is limited, as we did not collect qualitative data.

## 5 CONCLUSIONS

Ending HIV/AIDS as a public health threat by 2030 and achieving country ownership will require maximizing the use of existing healthcare delivery platforms and HIV prevention products [42]. Our study provides evidence for one potential path forward: allowing PEP and PrEP to be delivered at private pharmacies and giving PEP equal priority as other HIV prevention products. Pharmacies should be prioritized for demonstration studies and rollout to expedite implementation learnings, including the development of innovations that can help sustain pharmacy delivery at scale.

## COMPETING INTERESTS

PM is an employee of Novartis, outside of the present work. KN has received research funding from the Merck Investigators Studies Program. For the remaining authors, none were declared.

## AUTHORS’ CONTRIBUTIONS

KFO, EAB and KN contributed to the study conception and design of this pilot study. SDR and KFO led the development of the study protocol and data collection tools. VO, PM and PO led recruitment and study operations with support from JO, DW and KH. SDR, PB, MA and SG led data management. PB, SDR and KFO analysed the data. SDR wrote the first draft of the manuscript, and KFO provided senior author‐level feedback. All authors provided additional feedback, edits, and insights and approved the final manuscript for publication.

## FUNDING

The Pharm PrEP Pilot Extension Study was funded by the Gates Foundation (INV‐033052). KFO was additionally supported by the National Institute of Mental Health (R34 MH120106; R00 MH121166). JP and EAB were additionally supported by the Eunice Kennedy Shriver National Institute of Child Health and Human Development (R01HD108041). The funders had no role in the study design; collection, analysis or interpretation of the data, or writing of this manuscript.

## Supporting information




**Supporting Figure 1**. Modified delivery model.
**Supporting Figure 2a**. Prescribing checklist for initiation visits.
**Supporting Figure 2b**. HIV Risk Assessment Screening Tool (RAST) for PrEP initiation and continuation visits.
**Supporting Figure 2c**. Prescribing checklist for follow‐up visits.
**Supporting Figure 3**. PrEP and PEP initiations by day of week.
**Supporting Table 1**. Breakdown of positive STI testing results by client sex.

## Data Availability

The data that support the study findings are available on request from the corresponding author and not publicly available due to privacy or ethical restrictions.
