# Supplementary material for: A modified pharmacy provider‐led delivery model of oral HIV pre‐ and post‐exposure prophylaxis in Kenya: a pilot study extension
Source: J Int AIDS Soc. 2025 Jun 26;28(Suppl 1):e26467. doi: 10.1002/jia2.26467 (PMC12231645; doi:10.1002/jia2.26467)
Supplement: Supplementary file 1 — Supporting Figure 1. Modified delivery model. Supporting Figure 2a. Prescribing checklist for initiation visits. Supporting Figure 2b. HIV Risk Assessment Screening Tool (RAST) for PrEP initiation and continuation visits. Supporting Figure 2c. Prescribing checklist for follow‐up visits. Supporting Figure 3. PrEP and PEP initiations by day of week. Supporting Table 1. Breakdown of positive STI testing results by client sex. [file JIA2-28-e26467-s001.docx]

**Supporting Information for**

*“A modified pharmacy provider-led delivery model of oral HIV pre- and post-exposure prophylaxis in Kenya: a pilot study extension”*

Stephanie D. Roche, Victor Omollo, Peter Mogere, Magdalene Asewe, Stephen Gakuo, Preetika Banerjee, Kendall Harkey, Monisha Sharma, Jillian Pintye, Melissa Latigo Mugambi, Parth Shah, Josephine Odoyo, Patricia Ongwen, Daniel Were, Elizabeth A. Bukusi, Kenneth Ngure, Katrina F. Ortblad, on behalf of the Pharm PrEP Pilot Extension Study Team

**Supporting Figure 1**. Modified delivery model

Original care pathway for pharmacy-based PrEP delivery, with new implementation strategies detailed in yellow boxes.


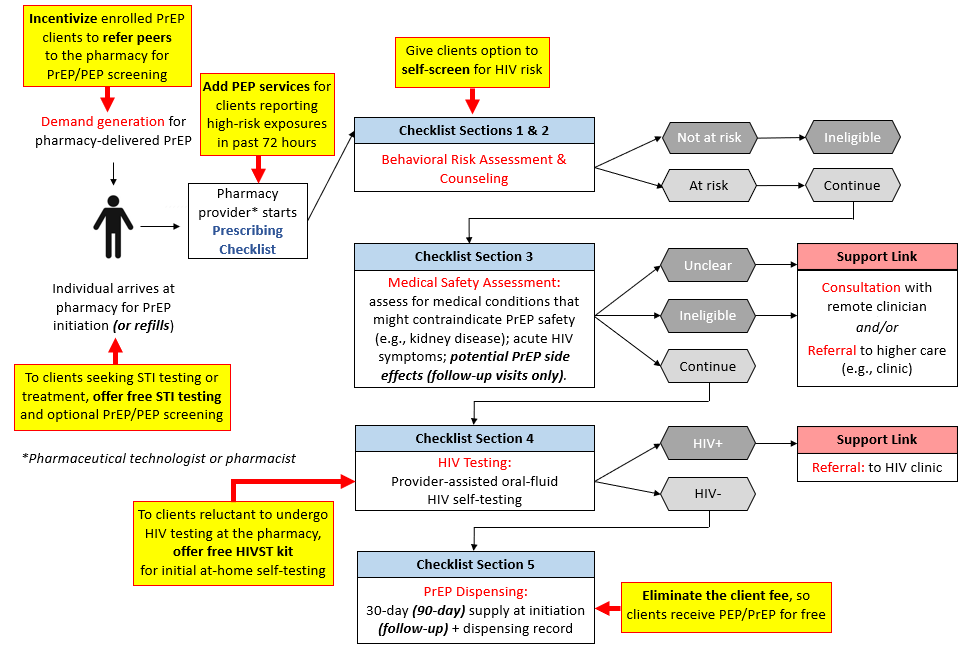


**Supporting Figure 2a.** Prescribing checklist for initiation visits


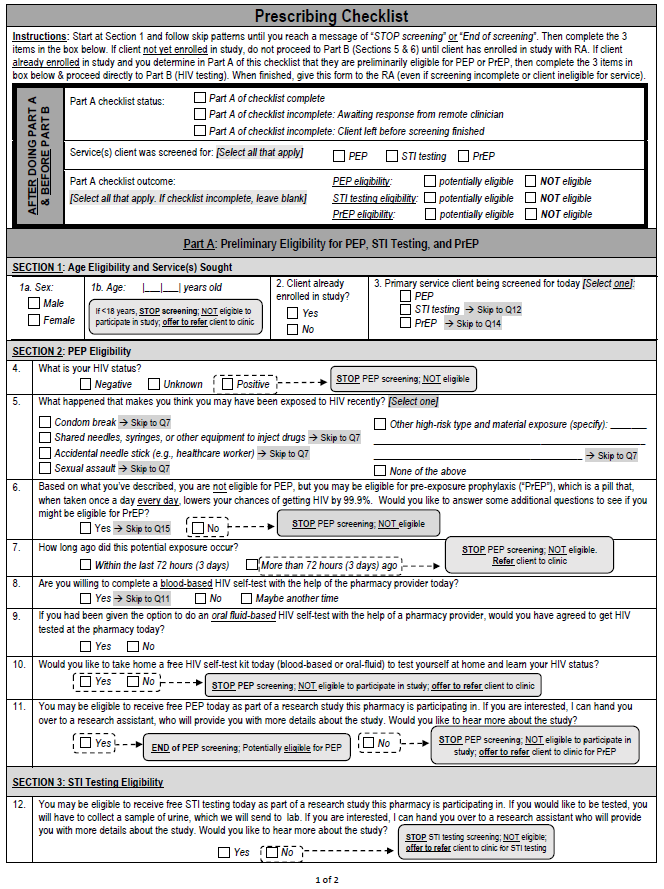


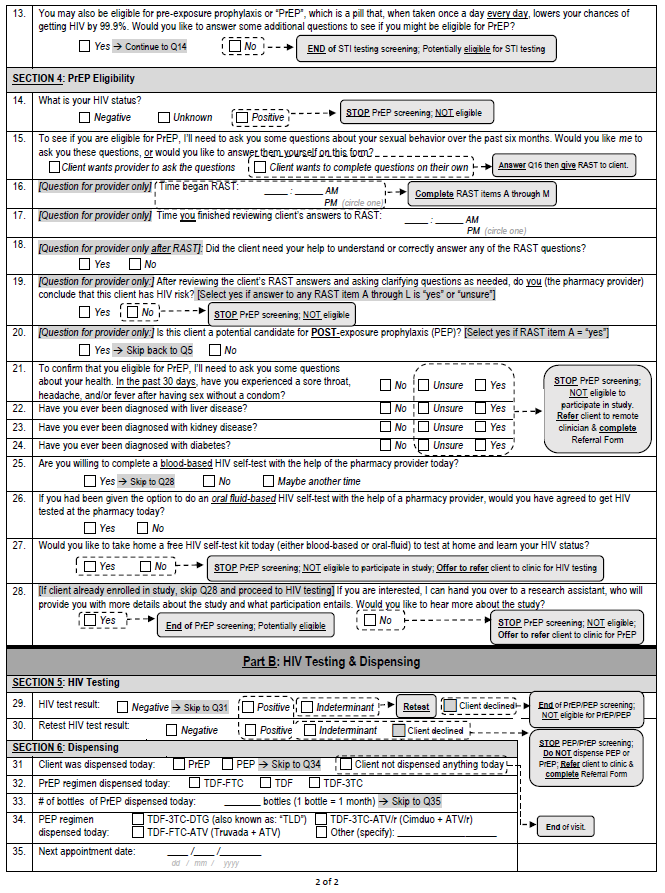


**Supporting Figure 2b.** HIV Risk Assessment Screening Tool (RAST) for PrEP initiation and continuation visits


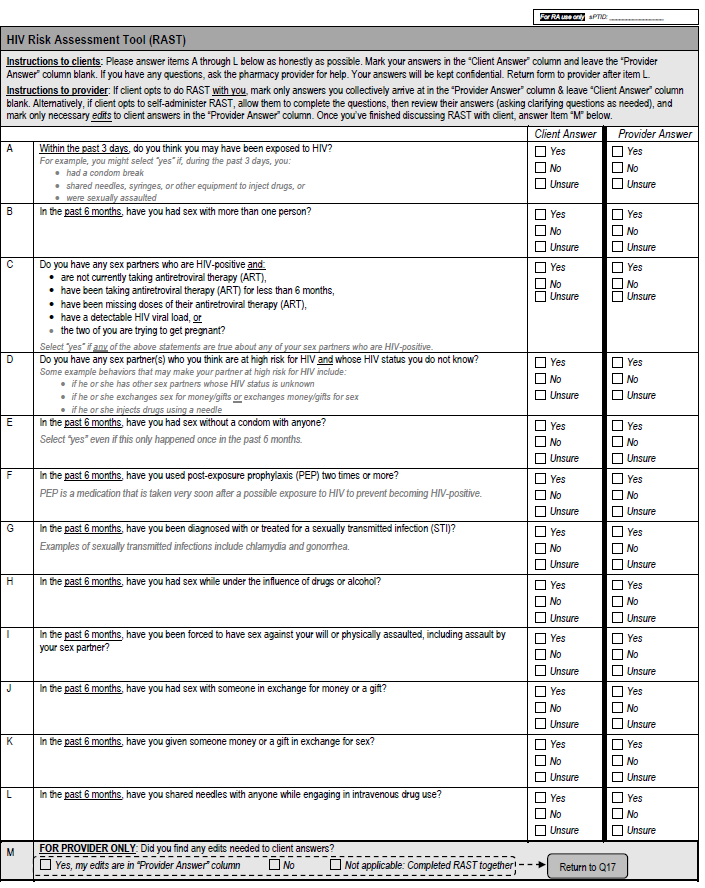


**Supporting Figure 2c.** Prescribing checklist for follow-up visits


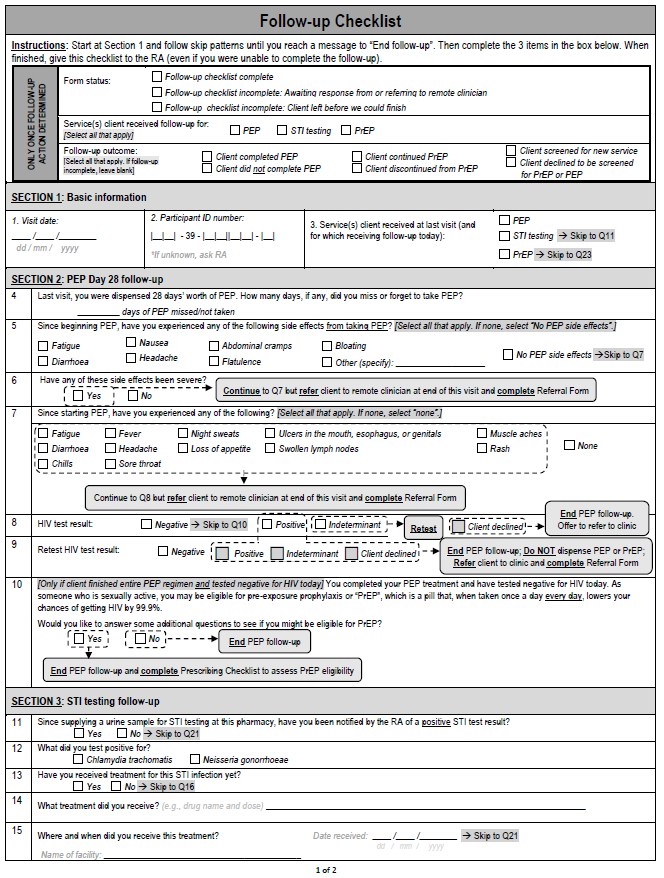


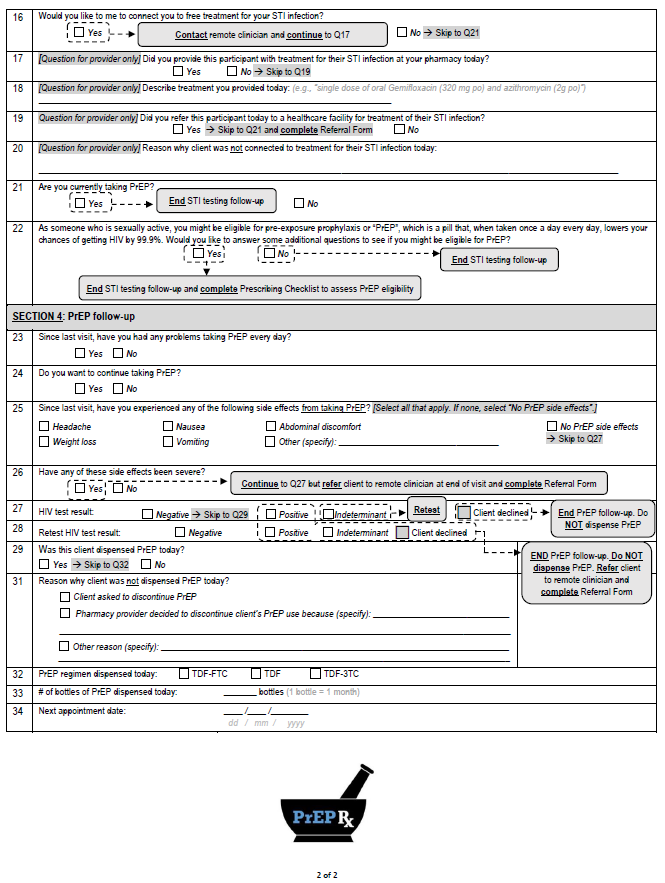


**Supporting Figure 3.** PrEP and PEP initiations by day of week

*Research assistants were not stationed at study pharmacies on Saturdays and Sundays, thus limiting new enrollments and PrEP/PEP initiations on these days.

**Supporting Table 1.** Breakdown of positive STI testing results by client sex

| **Test result** | **Females**  (N=6) | **Males**  (N=4) |
| --- | --- | --- |
| Positive for *N. gonorrhoeae* only | **3** | **2** |
| Positive for *C. trachomatis* only | **2** | **1** |
| Positive for both | **1** | **1** |
